# Migration, Work, and Health: Lessons Learned from a Clinical Case Series in a Northern Italy Public Hospital

**DOI:** 10.3390/ijerph16173007

**Published:** 2019-08-21

**Authors:** Cecilia Arici, Tishad Tamhid, Stefano Porru

**Affiliations:** 1 Department of Diagnostics and Public Health, Section of Occupational Health, University of Verona, 37134 Verona, Italy; 2 University Research Center “Integrated Models for Prevention and Protection in Environmental and Occupational Health”, Universities of Verona, Brescia and Milano Bicocca, 37134 Verona, Italy; 3 Postgraduate School of Occupational Medicine, University of Verona, 37134 Verona, Italy

**Keywords:** migrant workers, occupational diseases, health surveillance, fitness for work, clinical case series, occupational health services, Italy

## Abstract

Background: Migrant workers (MWs) generally perform dangerous jobs and have reduced access to occupational health (OH) care, therefore being prone to developing occupational diseases (OD). The aim of the work is to describe a case series of MWs and report on related outcomes for OH professionals. Methods: A case series of 724 MWs, sent from January 2001 to June 2013 to a public OH unit for OD or fitness-for-work (FFW) evaluation, was entered in a dedicated database and elaborated for descriptive statistics with Microsoft Excel. Results: MWs were mostly (75%) men, with a mean age of 40. They came mainly from Morocco, Senegal, Albania, Romania, and Pakistan. Main sectors of employment were manufacturing, metal industry, services, construction. OD were found in 210 cases, main diagnoses being: Lumbar disc and upper limb musculoskeletal disorders (51%), contact dermatitis (15%), allergic asthma (8%), noise-induced hearing loss (7%), tumors (3%), psychiatric disorders (2%). Moreover, 136 FFW judgements were formulated, with some limitations/restrictions expressed. Finally, a relevant prevalence of some chronic non-occupational diseases was found. Conclusions: MWs in Italy may suffer from OH inequalities. Qualified public OH professionals and occupational physicians in workplaces should have a proactive role to concretely meet MWs’ health needs.

## 1. Introduction

As broadly documented in the literature, on a worldwide basis migrant workers generally: Work for less pay, for longer hours, and in more precarious circumstances than non-migrants do; have higher rates of adverse occupational exposures and working conditions, which lead to poor health outcomes, workplace injuries, and occupational diseases; and are victims of relevant health disparities that are related to environmental and occupational exposures and are a result of language/cultural barriers, access to health care, documentation status, and the political climate of the host country [1,2,3,4,5,6,7,8].

It has been estimated that in Italy there are about 6 million international migrants that represent 10% of the national population [9], and about 2.5 million migrant workers (67% from non-EU countries, 33% from EU countries) that are mainly employed in health and social services (27%), industry (17%), and trade (11%) sectors [10,11]. As widely documented by the literature, migrant workers in Italy are mainly employed in manual, unqualified jobs that Italians tend not to perform anymore and tend to be more hired by precarious contracts and present a higher risk of work injuries and occupational illnesses than natives, mainly because of language and cultural barriers, greater risk tolerance, as well as a concentration of migrants in the so-called “3D jobs” (dangerous, dirty, and demanding/degrading) [12,13,14,15,16]. In particular, the available data from national official sources show a progressively increasing number of occupational diseases and work-related disorders among migrant workers in the last few years (i.e., from 1220 notified cases—4.6% of total—in 2004 to 3769 notified cases—6.5% of total—in 2017), with a sharply higher prevalence of musculoskeletal disorders among migrants from non-EU countries [14,15,16,17,18].

Despite such evidence, to the best of our knowledge (i.e., on the basis of searches in PubMed both with free terms and MeSH terms), there is not any peer-reviewed article that reports case series originated from clinical activities performed by public occupational health services/units. It should be noted here that in Italy, occupational medicine services/units cover both current and former (i.e., unemployed, retired) migrant workers with various employment conditions (e.g., employees, trainees/apprentices, atypical and self-employed workers), provided they are regular immigrants with compulsory occupational insurance coverage guaranteed by the Italian Workers’ Compensation Authority (INAIL). Migrant workers can be sent to such services/units by general practitioners, enterprises, occupational physicians, hospital wards and public institutions for the evaluation of occupational diseases and/or fitness-for-work judgement.

Therefore, the aims of the present work are:

➢ To present a case series stemming from an experience performed in a Northern Italy public occupational health service;
➢ To delineate areas of intervention and perspectives for occupational physicians and occupational health professionals, in order to guarantee access to occupational health services, as well as performances of good medical practices for risk assessment and health surveillance of migrant workers, and to prevent occupational health inequalities.

## 2. Methods

An experience of more than 10 years is reported, stemming from daily activities performed at a Northern Italy public Occupational Health Unit, located within a highly industrialized area (mainly metal and construction industries), where it is has been estimated that migrants represent 14% of the assisted population (i.e., about 170,000 units) [19].

Migrant workers were outpatients, usually sent for the evaluation of occupational diseases and/or fitness-for-work judgement, by general practitioners, enterprises, occupational physicians, hospital wards, and public institutions. Every migrant worker signed a detailed informed consent when starting the clinical assessments, enabling the clinicians to deal with the data. Then, each migrant worker underwent the following clinical procedure: History collection, including general demographic data, occupational exposure assessments, and clinical history; physical examination; instrumental/laboratory tests, chosen according to the relevant clinical questions; in some circumstances, documentation was acquired from general practitioners, enterprises and/or plant occupational physicians, local health authorities. At the end of the assessment, a detailed conclusive clinical report was sent to the worker, the practitioner, and the occupational physician.

In more detail, it was possible to enter in a dedicated Microsoft Access database the case series of all migrant workers evaluated from January 2001 to June 2013. These data were then elaborated for descriptive statistics, by means of Microsoft Excel.

## 3. Results

**Figure 1:**
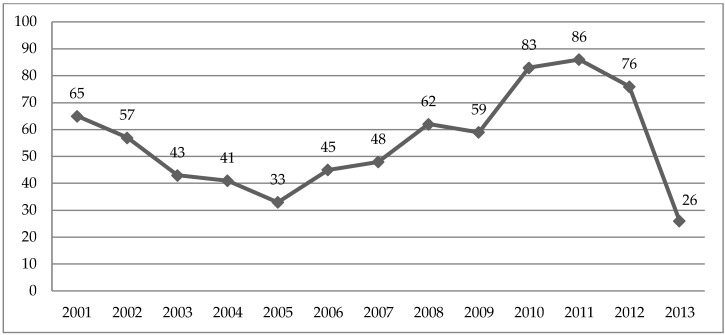
Number of migrant workers assessed at an occupational health public service from January 2001 to June 2013 (*n* = 724 subjects).

A total of 724 migrant workers were assessed, with an increasing trend between 2005 and 2011 (Figure 1).

They were mainly men (*n* = 542, 74.8%), resident in the province of Brescia (*n* = 605, 83.5%), aged on average 40.2 years (*n* = 162, 22.3% > 49 years old; *n* = 365, 50.4% between 35–49; *n* = 179, 24.7% between 18 and 34), with a medium/high level of education (34.9% secondary school degree, 36.0% high school degree, 6.6% university degree). As shown in Table 1, they came mainly from Morocco, followed by Senegal, Albania, Romania, and Pakistan, with an estimated mean duration of presence in Italy of 13.3 years (range 1–47, 55% more than 10).

With regard to their working conditions, they were mainly employed workers (*n* = 517, 71.4%), with a total mean duration of employment in Italy of 16.9 years (range 1–51, 36% between 11 and 20), mainly occupied in the manufacturing (27.0%) and metal industry (22.7%), followed by metal casting sector (14.0%), services (10.9%), and construction (7.5%). It should also be noted that 1.5% of them were self-employed workers. As for the remaining migrants, at the time of the assessment they were mainly unemployed (12.4%), trainees/apprentices (8.1%), atypical workers (3.8%) or retired (1.7%). Moreover, a sort of labour market segmentation was evident, with gender and nationality substantially influencing the type of job engaged: In fact, African and South American women typically worked as auxiliary health personnel; men from East Europe (in particular, Albanians, Romanians, Croatians, Serbians), North, Central, and West Africa (in particular, Moroccans, Senegalese, Egyptians, and Tunisians) were predominantly occupied in construction, metal, and manufacturing industry; Indians usually worked in agriculture.

As for the source of commitment, they were mainly sent by general practitioners (*n* = 398; 54.9%) and enterprises (*n* = 261; 36.0%), while the remaining subjects were sent by local health authorities or wards of the same public hospital.

**Figure 2:**
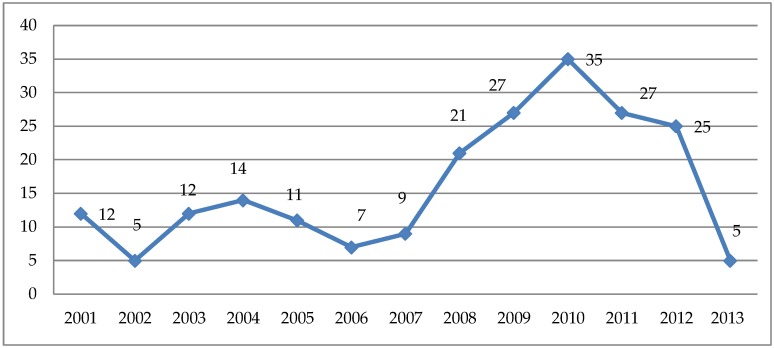
Occupational diseases/work-related disorders (*n* = 210) diagnosed among migrant workers assessed from January 2001 to June 2013.

When the source of commitment was the general practitioners, the main reason for evaluation was a suspected occupational disease/work-related disorder, in particular musculoskeletal disorders (25.9%), dermatitis (10.7%), allergic respiratory disorders (6.6%), tumours (4.0%), and hearing loss (3.3%). A total of 210 (29% of the whole case series) occupational diseases/work-related disorders were diagnosed, 76.1% in men and 23.8% in women, with an increasing trend between 2006 and 2010 (Figure 2). As shown in Table 2, such diseases/disorders were mainly represented by:

➢ Lumbar disc disease, generally affecting male migrant workers employed in metal casting sector, metal and manufacturing industry, as well as female migrants working as auxiliary personnel in the health care sector;
➢ Allergic contact dermatitis, mainly affecting construction and metal migrant workers;
➢ Upper limb musculoskeletal disorders, diagnosed in migrant workers employed in the manufacturing and metal industry;
➢ Asthma, particularly in foundry workers;
➢ Noise-induced hearing loss, prevalent in manufacturing industry male migrant workers.

In accordance with Italian legislation, all occupational diseases/work-related disorders were mandatorily notified to local health authority and reported to National Insurance Institute for workers’ occupational injuries and diseases (INAIL).

When the source of commitment was the enterprises, the main reason for evaluation was fitness for work. A total of 136 fitness-for-work judgements (52.1% of the whole case series) were formulated, with some limitations/restrictions expressed for some subjects. Table 3 reports the synopsis of the fitness-for-work assessments performed.

Finally, a relevant prevalence of some chronic non-occupational diseases was found among the 724 overall assessed migrant workers, in particular obesity (12.0%), hypertension (8.0%), cardiovascular diseases (3.0%; e.g., ischemic heart disease, valvulopathy, cardiomyopathy, and arrhythmias), diabetes (2.3%) and some, not otherwise specified, musculoskeletal degenerative disorders (3.1%).

## 4. Discussion

It is now common knowledge that work is certainly an important social determinant of health among migrant workers, who despite an often medium/high level of education generally have a poor and/or precarious position on the labor market and perform low-skilled and flexible jobs—mainly because of poor language skills, poor knowledge of the labor market, less efficient job-finding strategies than native workers, difficulties in validating original qualifications—therefore being exposed to disparities in work-related exposures that, in turn, lead to higher rates of diseases [1,2,3,4,5,6,7,8]. However, to the best of our knowledge, this is the first peer-reviewed article reporting a general case series of migrant workers analyzed from the experience of an occupational health public service.

In accordance with previous literature [12,13,15], a labor market segmentation was evident in our experience. In comparison with national official statistics [17,18], we found: A similar prevalence of work-related musculoskeletal disorders (55.1% vs. 55.9%), noise-induced hearing loss (6.6% vs. 6.4%), and tumors (2.8% vs. 2.9%); a relatively high prevalence of occupational skin diseases (18.4% vs. 16.8%) and respiratory disorders (14.2% vs. 13.3%). This means sort of a selection of the migrant population for certain diseases, but it reflects more accurately the real world found in occupational health settings. Moreover, our experience documented a relevant prevalence of a number of non-occupational diseases (e.g., musculoskeletal degenerative disorders, obesity, diabetes, hypertension, and cardiovascular diseases). This is particularly relevant—in fact, there is evidence that international migrants are not a random sample from their home countries, since they are “positively selected” (i.e., the so-called “healthy migrant” effect); however, over time the newcomers’ health advantages diminish and their health status increasingly resembles that of natives; therefore, it is to be expected to diagnose the same diseases affecting natives [1,2,3,4,5,6,7,8].

It should be here underlined that many of both the occupational diseases (e.g., back and upper limb musculoskeletal disorders, contact dermatitis, asthma, noise-induced hearing loss, psychiatric disorders) and the chronic non-occupational diseases (e.g., obesity, hypertension, diabetes) found in our case series had never been diagnosed and/or managed before, either at work or outside. Therefore, migrant workers seem to require a greater attention than the natives because of a high level of unmet health needs, which can increase individual vulnerability and susceptibility to occupational hazards, therefore requiring focused case-management approach within the occupational health settings. Moreover, the type of limitations and/or prescriptions expressed in fitness-for-work evaluations reflect what expected from the main diagnostic questions and foster preventive actions at primary, secondary and tertiary levels. Therefore, occupational health services represent an important and unique opportunity for conserving and improving migrant workers’ health, given that focused health surveillance, fitness-for-work evaluation, and health promotion must be performed by a qualified, accountable, and motivated occupational physician.

One possible limitation of the study which should be acknowledged is that the case series presented here refers to the period 2001–2013, and thus it could be not representative of the current Italian socio-economic and occupational situation. However, on the basis of the daily activities that are now performed in Northern Italy public Occupational Health Units, as well as according to updated information from national official sources [10,11,17,18], we are reasonably certain that relevant socio-economic or occupational changes have not occurred in the meantime; moreover, the Italian public health system has remained unchanged. Other possible limitations are that the present data are not representative of the occupational health status of migrant workers in the shadow labor market and that self-employed migrants represented only 1.5% of the total case series. Finally, the results presented here cannot be generalized to the total migrant labor force, since the sample was not selected randomly.

Therefore, the main implications and advantages stemming from the study, in our opinion still representative of the status quo, are the following:

➢ The evidence of an important role played by occupational health public services;
➢ The need for second level diagnosis;
➢ The response to migrant workers’ health needs, by means of focused and multidisciplinary risk assessment, health surveillance, fitness for work, case management, and health promotion;
➢ The collaborative dialogue with practitioners, especially for non-occupational diseases;
➢ The characterization of migrant workforce;
➢ More social protection for and more opportunities for compensation for work-related disorders in migrant workers.

## 5. Conclusions

The occupational health community is increasingly turning its attention to the effects of work on migrant workers, and researchers have identified examples of disparities in occupational health outcomes [1,2,3,4,5,6,7,8].

Migrant workers in Italy may suffer from occupational health and safety inequalities and may present unrecognized and underestimated general health impairments [10,11,12,13,14,15,16,17,18].

In our opinion, there is an urgent need to enhance and improve occupational health surveillance of migrant workers, in order to better describe nature and extent of disparities in occupational diseases and injuries, identify priorities for research and intervention, and evaluate trends. Moreover, the occupational health and safety training of migrant workers should also be improved, taking into appropriate consideration cultural and language issues.

Our study partially addresses these needs, providing detailed information about migrant workers’ occupational health status, with focus on various Italian sectors and multiple occupational hazards (chemical, physical, ergonomic, and psychosocial/organizational). Moreover, the study subjects came from many different countries, located in Africa, Europe, Asia, and America, thus allowing a multicultural approach to data collection and analysis.

In conclusion, occupational health professionals should be encouraged to play a proactive role in workplaces, aimed at real integration of migrant workers, in order to reach benefits for workers, enterprises, and society.

## Figures and Tables

**Table 1:** Assessed migrant workers’ main countries of origin.

Country of Origin	Number	%
Morocco	145	20.0
Senegal	64	8.8
Albania	63	8.7
Romania	43	5.9
Pakistan	42	5.8
India	31	4.3
Former Yugoslavia	30	4.1
Tunisia	29	4.0
Egypt	28	3.9
Ghana	27	3.7
Switzerland	19	2.6
France	15	2.1
Nigeria	13	1.8
Bangladesh	12	1.7
Poland	10	1.4
Ukraine	10	1.4
Libya	9	1.2
Brazil	9	1.2
Algeria	8	1.1
Argentina	7	1.0
China	7	1.0
Ivory Coast	7	1.0
Other	96	13.3
Total	724	100.0

**Table 2:** Main types of occupational diseases/work-related disorders diagnosed among the assessed migrant workers.

Diagnosis	No. of Cases (Prevalence)	Prevalence among Cases Notified to Italian Workers’ Compensation Authority (Inail)
Degenerative lumbar disc disease	75 (35.7%)	Musculoskeletal disorders 55.9%
Upper limb musculoskeletal disorders (including carpal tunnel syndrome)	32 (15.2%)	
Other musculoskeletal disorders	9 (4.2%)	
Allergic contact dermatitis	26 (12.3%)	Skin diseases 16.8%
Irritant contact dermatitis	7 (3.3%)	
Other skin diseases	6 (2.8%)	
Asthma	16 (7.6%)	Respiratory disorders 13.3%
Other respiratory disorders (e.g., rhinitis, chronic obstructive pulmonary disease—COPD)	14 (6.6%)	
Noise-induced hearing loss	14 (6.6%)	6.4%
Tumours	6 (2.8%)	2.9%
Psychiatric disorders	5 (2.3%)	2.4%

**Table 3:** Synopsis of the fitness-for-work evaluations performed among migrant workers from January 2001 to June 2013.

Fit	Fit with Prescriptions/Limitations		Unfit	Total
101 (74.2%)	31 (22.7%)		4 (2.9%)	136
	NUMBER (%)	MAIN REASONS FOR/TYPES OF		
	14 (45.1)	back and upper limb musculoskeletal disorders (heavy manual labor, awkward postures, manual materials handling)		
	3 (9.6)	shift work		
	3 (9.6)	respiratory disorders		
	2 (6.4)	skin diseases		
	1 (3.2)	vision impairment		
	1 (3.2)	psychiatric disorder		
